# Rapid clearance of *Schistosoma mansoni* circulating cathodic antigen after treatment shown by urine strip tests in a Ugandan fishing community – Relevance for monitoring treatment efficacy and re-infection

**DOI:** 10.1371/journal.pntd.0006054

**Published:** 2017-11-13

**Authors:** Anna O. Kildemoes, Birgitte J. Vennervald, Edridah M. Tukahebwa, Narcis B. Kabatereine, Pascal Magnussen, Claudia J. de Dood, André M. Deelder, Shona Wilson, Govert J. van Dam

**Affiliations:** 1 Section for Parasitology and Aquatic Pathobiology, Faculty of Health and Medical Sciences, University of Copenhagen, Copenhagen, Denmark; 2 Vector Control Division, Ministry of Health, Kampala, Uganda; 3 Schistosomiasis Control Initiative, Ministry of Health, Kampala, Uganda; 4 Centre for Medical Parasitology, Faculty of Health and Medical Sciences, University of Copenhagen, Copenhagen, Denmark; 5 Department of Molecular Cell Biology, Leiden University Medical Center, Leiden, The Netherlands; 6 Department of Parasitology, Leiden University Medical Center, Leiden, The Netherlands; 7 Department of Pathology, University of Cambridge, Cambridge, United Kingdom; Swiss Tropical and Public Health Institute, SWITZERLAND

## Abstract

Schistosomiasis control and elimination has priority in public health agendas in several sub-Saharan countries. However, achieving these goals remains a substantial challenge. In order to assess progress of interventions and treatment efficacy it is pertinent to have accurate, feasible and affordable diagnostic tools. Detection of *Schistosoma mansoni* infection by circulating cathodic antigen (CCA) in urine is an attractive option as this measure describes live worm infection noninvasively. In order to interpret treatment efficacy and re-infection levels, knowledge about clearance of this antigen is necessary. The current study aims to investigate, whether antigen clearance as a proxy for decreasing worm numbers is reflected in decreasing CCA levels in urine shortly after praziquantel treatment. Here CCA levels are measured 24 hours post treatment in response to both a single and two treatments. The study was designed as a series of cross-sectional urine and stool sample collections from 446 individuals nested in a two-arm randomised single blinded longitudinal clinical trial cohort matched by gender and age (ClinicalTrials.gov Identifier: NCT00215267) receiving one or two praziquantel treatments. CCA levels in urine were determined by carbon-conjugated monoclonal antibody lateral flow strip assay and eggs per gram faeces for *S*. *mansoni* and soil-transmitted helminths by Kato-Katz. Significant correlations between CCA levels and *S*. *mansoni* egg count at every measured time point were found and confirmed the added beneficial effect of a second treatment at two weeks after baseline. Furthermore, presence of hookworm was found not to be a confounder for CCA test specificity. Twenty-four hours post treatment measures of mean CCA scores showed significant reductions. In conclusion, removal of CCA in response to treatment is detectable as a decline in CCA in urine already after 24 hours. This has relevance for use and interpretation of laboratory based and point-of-care CCA tests in terms of treatment efficacy and re-infection proportions as this measure provides information on the presence of all actively feeding stages of *S*. *mansoni*, which conventional faecal microscopy methods do not accurately reflect.

**Trial registration:**

ClinicalTrials.gov NCT00215267

## Author summary

Large scale efforts to control schistosomiasis in several sub-Saharan countries are in progress. In order to accurately monitor the effect of interventions, we need diagnostic tools which are highly specific, sensitive, affordable and easy to use and implement. For *Schistosoma mansoni* detection the circulating cathodic antigen (CCA) is an attractive option as this antigen is a measure of actively feeding worms and can be measured in urine samples. However, knowledge about how fast this antigen is cleared in response to treatment with praziquantel is necessary for interpretation of consecutive measures based on CCA in order to use this tool optimally. Here we show that CCA is already significantly reduced 24 hours after treatment both in single and double treatment regimens in a community sample from Musoli Village, Uganda. Furthermore, the data supports interpretation of trace measures of CCA as positive since the majority of individuals with trace measures respond to treatment. These observations provide a basis for extended use of CCA-based tools in monitoring treatment efficacy and possibilities for logistically advantageous prevalence screening strategies.

## Introduction

The ambitious London declaration prompted by the WHO 2012 roadmap to combat neglected tropical diseases commits to global schistosomiasis control by 2020 [1, 2]. Mass drug administration with praziquantel is the most widely implemented intervention for control. However, the necessity of an inter-disciplinary approach incorporating other strategies, such as community health education, improved safe water supply, sanitation and control of intermediate hosts, has become evident for targeting elimination of this poverty associated disease [3]. For elimination strategies, where low infection intensities and focal epidemiology must be addressed in the later stages of a control programme, a test-then-treat approach based on point-of-care tests, preferably incorporated in integrated disease control programmes, becomes relevant in contrast to continuous reliance on mass drug administration only protocols.

In order to accurately monitor the progress of interventions aiming at reducing, controlling or even regionally eliminating transmission, we need highly specific, sensitive, feasible and affordable diagnostic tools, which optimally live up to all the ASSURED criteria (Affordable, Sensitive, Specific, User-friendly, Rapid and robust, Equipment-free and Deliverable to end-users) [4, 5]. *Schistosoma* spp. worm antigens, which are released by living worms, are attractive targets compared to specific antibodies as they enable identification of currently infected individuals compared to individuals with previous infection. This is very relevant for assessing treatment efficacy as praziquantel kills adult schistosomes and mature eggs, but does not affect the immature worm and egg stages [6–8].

Circulating cathodic antigen (CCA), which is a mucin-type glycoprotein antigen [9], regurgitated by *Schistosoma* spp. [10–14], has been shown to be a good measure for active *Schistosoma mansoni* infection [15]. CCA is excreted into the bloodstream as soon as schistosomules start actively feeding and at least in high dose mouse models the antigen level is positively correlated to worm burden [16]. In humans the earliest verifiable detection point so far demonstrated is four weeks post infection [17]. In addition, CCA can be detected in sera and breast milk from infected individuals [18]. CCA detectable in human urine was first described by Carlier *et al*. (1975) and Deelder *et al*. (1976) [11, 19]. However, the potential of this antigen was not recognised until recently [20].

Two robust immunochromatographic methods for direct CCA detection in human urine are now published; a laboratory based strip test (using colloidal carbon labelling) and a commercially available point-of-care test (POC-CCA, Rapid Medical Diagnostics). Rapid antigen clearance after praziquantel treatment is essential for CCA-based diagnostic tools to be used for interpreting treatment efficacy, re-infection dynamics and potential development of drug resistance. Furthermore, knowledge of antigen clearance is very relevant for identifying target groups harbouring persistent active infection such as egg negative individuals. In this study the laboratory based lateral flow urine strip test was used to investigate whether changes in CCA score were detectable as early as 24 hours after treatment in urine samples from a community living in a *S*. *mansoni* high endemic area by Lake Victoria, Uganda. Furthermore, the relationship between *S*. *mansoni* egg output and CCA score in relation to age groups and responses to a single versus double treatment schedules were assessed.

## Materials and methods

### Study design

**Fig 1:**
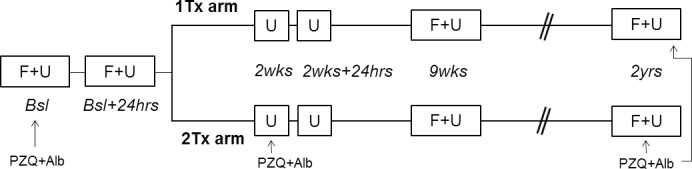
Schematical overview of sampling time points and treatment regimens. Diagram showing the faecal (F) and urine (U) sample collection time points included in this study; baseline (bsl), baseline+24hours (bsl+24), two weeks (2wks), two weeks+24hours (2wks+24hrs), nine weeks (9wks) and two years (2yrs). Both praziquantel (PZQ) and albendazole (Alb) was administered to everyone at baseline and the end of the study (2yrs). An additional dose of PZQ was administered at the two week time point to the two treatments arm (2 Tx).

The current study is based on a series of cross-sectional sampling points nested in a two-arm cohort randomised single blinded longitudinal clinical trial on “The effect of praziquantel treatment on *Schistosoma mansoni* morbidity and re-infection along Lake Victoria, Uganda” (ClinicalTrials.gov Identifier: NCT00215267). A questionnaire based census formed the basis for random recruitment of participants above seven years of age stratified by gender and age [21, 22]. Sample size for the clinical trial set-up was estimated to 552 individuals using cure rate data from Lake Albert, Uganda [23] with a power of 90%, significance level of α = 0,05 and an anticipated drop-out from baseline (2005) to finalisation (2007) of 40%. Randomisation to either the arm receiving one dose of praziquantel treatment (1 Tx) or the arm receiving a second dose two weeks (2 Tx) after baseline was done by a scientist not involved in treatment and laboratory work using computer generated random numbers [24]. The study was blinded for examiners, but participants were fully informed of treatment regimen. Urine samples were investigated at five time points and stool samples at two time points after baseline (Fig 1). Sample sizes for each analysis are stated; no-show or difficulty delivering stool/urine for individuals at some time points account for missing data (S1 Dataset).

### Study area and population

Study participants were recruited from Musoli Village, Mayuge District in south-east Uganda, which is located along Lake Victoria. Mayuge District is located 1161m above sea level and receives annual precipitation ranging from 600-1100mm with average temperatures from 19–27°C [25]. This area of Lake Victoria displays high perennial *S*. *mansoni* transmission and no *S*. *haematobium* transmission (personal communication N. Kabatereine). The only water source available to Musoli Village for both domestic and recreational use was Lake Victoria, hence exposing the population to schistosomiasis. Albeit, that Mayuge District authority had implemented praziquantel mass drug administration for control purposes, Musoli Village had not yet received treatment at the time of the study. Besides fishing, the local economy relies on subsistence farming.

### Ethical considerations

Ethical approval was obtained from the Higher Degrees Research and Ethics Committee of the School of Public Health, Makerere University. Ethical clearance was granted by the Uganda National Council of Science and Technology (Reference number: UNCST:HS59).

Informed consent written in Lusoga, which is the most commonly used local language, was obtained from each adult participant. Assent was obtained from participants under 15 years of age, which also were required to present a signature from a parent or guardian. Participants were free to withdraw from the study at any point in time without facing any form of repercussion.

All participants were treated with standard 40mg/kg single dose praziquantel (Distocide 600mg, Shin Poong Pharmaceuticals) at baseline irrespective of infection status. Furthermore, 400mg of albendazole (Alzental 400mg, Shin Poong Pharmaceuticals) was given to each participant for treatment of soil-transmitted helminths. One participant group received a second standard single dose of praziquantel (40mg/kg) two weeks after baseline. Before treatments all participants were offered a snack and a drink in order to minimise adverse events. After treatment participants were observed for two hours for management of possible adverse events. An experienced nurse directly observed all treatments [24]. After finalisation of the clinical trial in 2007, the whole community including the study participants were treated with a single standard dose of both praziquantel and albendazole following national guidelines.

### Parasitology

#### Urine samples

Morning to mid-day urine samples [26] for analysis of CCA were collected at six time points; baseline, baseline +24hrs, two weeks, two weeks +24hrs, nine weeks and two years. Single urine samples were collected from participants and kept cold, swirled and aliquoted before being frozen at -20°C for storage until analysis. CCA is a very temperature stable glycoprotein and read-outs are not affected by multiple freeze-thaw cycles, prolonged freezing at only -20°C or even storage at room temperature for up to weeks (personal communication G. J. Van Dam). The immunochromatographic carbon-conjugate based CCA urine strip assay was performed as previously described [27, 28]. This lateral flow urine strip method is based on CCA-detection by IgG monoclonal antibodies (IgG1 54-4C2-A and IgG3 54-5C10-A [29]) and includes a positive control of polyclonal anti-mouse antibodies, which catch excess carbon-conjugated antibodies. Briefly, 25ul urine was added to a tube containing dried carbon conjugated antibody, along with 75uL of buffer and mixed well. Test strips were added, and allowed to develop for 40 minutes after which the strips were removed. They were read dry and colour reaction intensity was interpreted by comparison to a dilution standard series of semi-purified *S*. *mansoni* worm antigen containing 3% CCA (0, 10, 100, 1000, 10000ng/ml). Read-outs ≥100ng/ml were considered positive and assigned a positive score (logarithmic scale) related to increasing colour intensity (score 0, ½, 1, 2, 3 respectively). Score 0 was assigned to negative strip readings. A trace measure of 0,5 was given to strip readings of lower intensity than 100ng/ml. Strips were read by two independent experienced researchers in a blinded manner. If score discrepancies were found, a third experienced researcher was consulted to conclude on the score.

#### Stool samples

Early morning stool specimens were collected on three consecutive days from all participants. Two slides from each stool specimen were prepared using the modified Kato-Katz (KK) thick smear method on 50mg templates [30]. The six slides per participant were examined by experienced technicians using light microscopy at 10x magnification. The slides were read within one hour for hookworm egg presence and ~24 hours after preparation for *S*. *mansoni*, *Ascaris lumbricoides* and *Trichuris trichiura*. 10% of the slides were re-examined by an independent experienced microscopist for quality control purpose. Variations in *S*. *mansoni* egg counts by quality control were less than 5% (B. J. Vennervald personal communication). *S*. *mansoni* infection intensity was categorised as low (1–99 eggs per gram (EGP)), moderate (100–399 EPG) and heavy (≥400 EPG) [31]. Stool investigation was performed at baseline, after nine weeks and two years as described by Tukahebwa *et al*. (2013).

### Data management and statistical analyses

All data was double-entered in Microsoft Excel 2003 spreadsheet software, where after the data file was exported to SPSS (IBM) for further analyses. Graphical illustrations were made using GraphPad Prism 6 software. For all statistical analyses p values of <0.05 were considered significant and two tailed tests applied. Hookworm species, *A*. *lumbricoides* and *T*. *trichiura* results were only recorded as positive/negative during data collection. Spearman’s correlation co-efficient (*rho*) was used to describe relationships between CCA score and KK egg intensity. Kruskall-Wallis/Mann-Whitney was used to compare mean EPG for 6KK/individual slide EPG and CCA score stratified by treatment regimen and time points. Mann-Whitney was also used to investigate associations between CCA and hookworm status. Paired t-tests were applied for comparing the means of CCA scores with 24 hour intervals. Binary logistic regression models were used to assess the odds for being CCA positive when positive for *S*. *mansoni* (controlled for hookworm, gender and child/adult) or hookworm eggs.

## Results

The total number of recruited participants with CCA and *S*. *mansoni* egg count data was 446 individuals, of which 228 (51,1%) were male and 218 (48,9%) female, originating from 243 different households. The median age was 23 years (range 7–76 years). Participants were divided in two treatment arms stratified by gender and age; 240 individuals received praziquantel only at baseline while the other arm of 206 individuals received a second dose 2 weeks after baseline. Demographic, occupational, *S*. *mansoni* morbidity and infection intensity (KK) data including dose related treatment effect were reported in Tukahebwa *et al*. 2013 and Koukounari *et al*. 2013 [21, 22]. Prevalence of *S*. *mansoni* infection for each sampling time point based on detection of CCA in urine (trace -/+) or eggs in faeces is shown in Table 1.

**Table 1:** *S*. *mansoni* prevalence (%) measured by urine strip CCA or KK for both treatment arms over time.

	Sampling timepoint:	Bsl	Bsl+24hrs	2 wks	2wks+24hrs	9 wks	2 yrs
CCA	Prevalence Tx1 (n)—Tr-	76% (238)	52% (212)	44% (202)	50% (151)	50% (211)	72% (170)
	Prevalence Tx1 (n)—Tr+	80% (238)	63% (212)	54% (202)	56% (151)	60% (211)	76% (170)
	Prevalence Tx2 (n)—Tr-	80% (206)	48% (180)	41% (205)	28% (158)	32% (188)	67% (156)
	Prevalence Tx2 (n)—Tr+	84% (206)	56% (180)	58% (205)	44% (158)	44% (188)	74% (156)
1KK	Prevalence Tx1 (n)	80% (236)	n/a	n/a	n/a	30% (224)	54% (185)
	Prevalence Tx2 (n)	83% (206)	n/a	n/a	n/a	17% (196)	56% (160)
3KK	Prevalence Tx1 (n)	89% (239)	n/a	n/a	n/a	48% (228)	67% (186)
	Prevalence Tx2 (n)	88% (206)	n/a	n/a	n/a	31% (200)	68% (160)

One KK prevalence is based on data from day 1 KK (Table A in S1 Table: KK slide 1.1 and 1.2). Three KK includes all available KK data from each person (from 1 to 3 KKs, 1–6 slides). Sample size (n) is given in parentheses. “Tx” = treatment; “Tr-”CCA score 0.5 considered negative; “Tr+” CCA score 0.5 considered positive; “n/a” = not applicable.

In the total sample, the cured proportions determined based on CCA in urine at baseline (trace considered positive) are 32,3% (104/322; trace = positive) and 41,0% (132/322; trace = negative) at baseline +24 hours and 36,3% (121/333; trace = positive) and 50,5% (168/333; trace = negative) at two weeks. For two weeks +24 hours the cured proportions are 50,0% (67/134; trace = positive) and 66,4% (89/134; trace = negative) in the two treatments arm compared to baseline (trace considered positive). The one treatment group at the two week time point show cured proportions in response to a single treatment at baseline of 36,4% (43/118; trace = positive) and 43,2% (51/118; trace = negative). Correspondingly, when considering trace as negative at baseline, the following cured proportions were observed; baseline +24 hours 30,0% (92/307; trace = positive) and 38,4% (118/307; trace = negative), two weeks 34,9% (111/318; trace = positive) and 48,7% (155/318; trace = negative), and for the two treatments arm at two weeks +24 hours 47,2% (60/127; trace = positive) and 64,6% (82/127; trace = negative). For the one treatment arm the cured proportions are 34,2% (39/114; trace = positive) and 41,2% (47/114; trace = negative) when assuming trace is negative for baseline measures.

### CCA and egg measures (baseline, nine weeks and two years)

**Fig 2:**
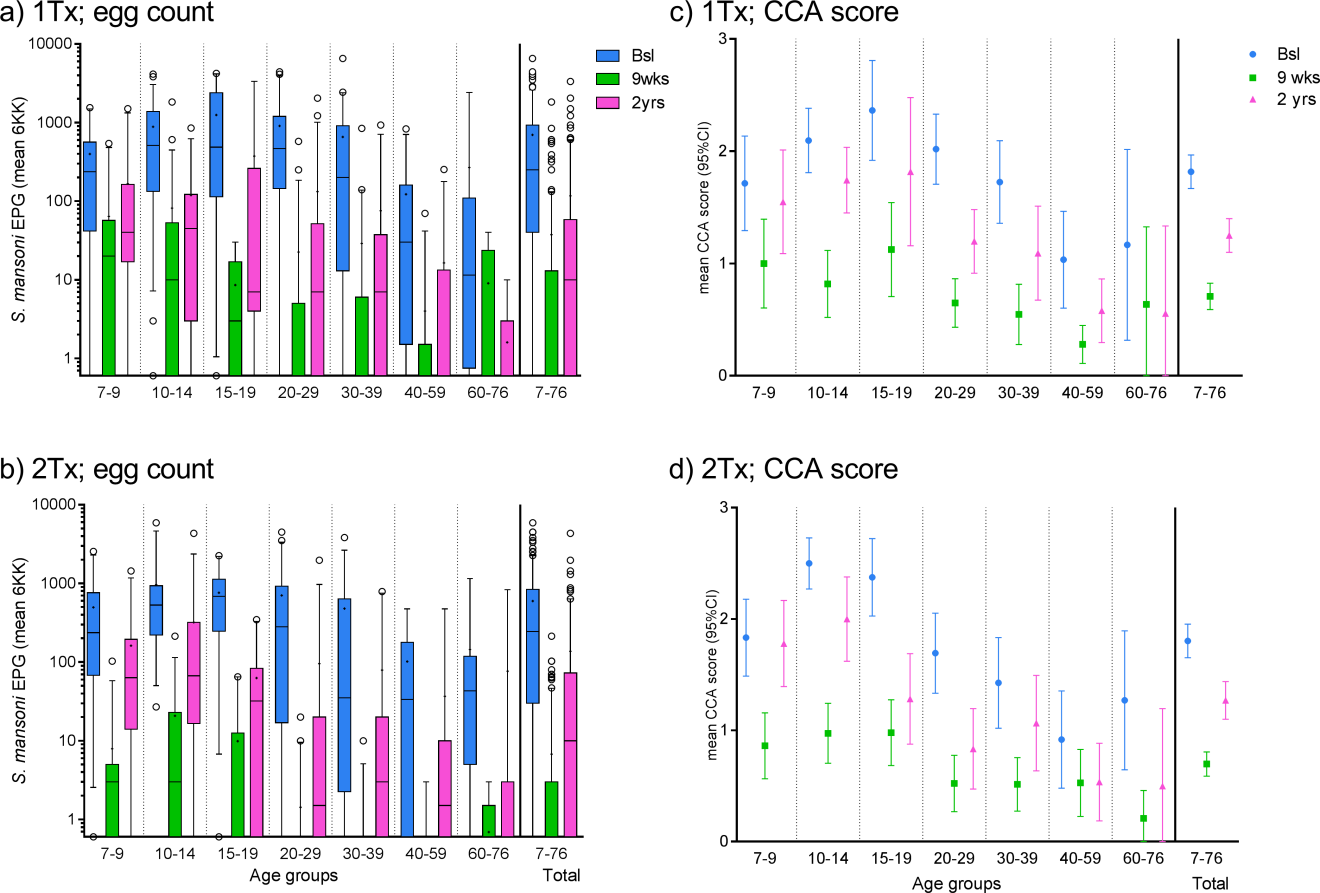
*S*. *mansoni* egg counts and CCA scores stratified by age groups and treatment arms. a) and b) Boxplot with whiskers (5-95th percentile) showing *S*. *mansoni* EPG (mean of all obtained KK/individual) related to participant age for a) the one treatment arm (n_bsl_ = 239, n_9wks_ = 228, n_2yrs_ = 186) and b) the two treatments group (n_bsl_ = 206, n_9wks_ = 200, n_2yrs_ = 160) at three time points. Circles depict outliers. Medians are indicated with horizontal bars and the means with small “+”. The top and bottom of the box represent the 75th and 25th percentile respectively. c) and d) show mean CCA score ±95% CI stratified by age groups for c) the one treatment arm (n_bsl_ = 238, n_9wks_ = 211, n_2yrs_ = 170) and d) the two treatments arm (n_bsl_ = 206, n_9wks_ = 188, n_2yrs_ = 156) at three time points. The distribution for all samples per time point is shown at the far right on all subfigures.

CCA score (0, ½, 1, 2, 3) and *S*. *mansoni* eggs (EPG) were both obtained at baseline, nine weeks and two years. There was no significant differences between treatment arms for CCA scores or egg intensities at baseline (CCA p = 0,792, n = 444; eggs p = 0,553, n = 445) or two years after treatment (CCA p = 0,951, n = 326; eggs p = 0,716, n = 346). However, significantly lower CCA scores (p = 0,001, n = 399) and fewer eggs (p<0,001, n = 428) were observed at nine weeks in the two treatments arm compared to the one treatment arm. For *S*. *mansoni* egg positive participants the geometric mean egg count was 253 EPG; 95% CI[212–301] at baseline (n = 395); 15 EPG, 95% CI[12–19] at nine weeks (n = 170) and 41 EPG; 95% CI[33–52] at two years (n = 232). *S*. *mansoni* prevalence and geometric mean egg counts for each KK slide and 6KK at baseline, 9 weeks and 2 years stratified by treatment regimen can be found in Table A in S1 Table. The arithmetic mean CCA score for positive participants were 2,2; 95% CI[2,1–2,3] at baseline (n = 365), 1,3; 95% CI[1,2–1,4] at nine weeks (n = 210) and 1,7; 95% CI[1,6–1,8] at two years (n = 246) (trace considered positive). CCA data is restricted to a narrow range of defined assay band intensity related observations (0–3), which limits demonstration of potential variation and average measures might not be truly representative, but they are used here in lack of a better method of illustration. Arithmetic mean CCA score and geometric mean egg counts for the three time points stratified by age groupings and treatment regimens are shown in Fig 2.

Positive correlations between CCA score and *S*. *mansoni* egg intensity were found at all measured time points (see Table B in S1 Table for CCA score/egg intensity category distribution data). The correlation was stronger at baseline (p<0,001, n = 443, *rho* = 0,717) and after two years (p<0,001, n = 320, *rho* = 0,691) than at nine weeks (p<0,001, n = 395, *rho* = 0,525). Similar findings are obtained when stratifying according to treatment regimen with better correlation at two years post treatment for both the two treatments arm (p<0,001, n = 153, *rho* = 0,762) and one treatment arm (p<0,001, n = 167, *rho* = 0,611) than at nine weeks for two treatments (p<0,001, n = 185, *rho* = 0,430) and one treatment (p<0,001, n = 210, *rho* = 0,554).

### Soil transmitted helminths (baseline, nine weeks and two years)

The prevalence of *A*. *lumbricoides* was very low. Only one individual was found infected at baseline (n = 430), none at 9 weeks (n = 426) and at two years a different single egg positive individual was found (n = 341). No individuals were at any time point found to be *T*. *trichiura* egg positive. The hookworm species prevalence was 44,3% (193 of 436) at baseline, 5,6% (24 of 425) at nine weeks and 28,0% (97 of 346) at two years. Of the individuals with hookworm data both at baseline and nine weeks, 180 were positive at baseline of which 20 remained positive at nine weeks. Of these thirteen individuals remained hookworm infected at two years post treatment (76,5%, n = 17).

Among *S*. *mansoni* egg negative individuals there was no association between hookworm and being CCA positive at baseline (p = 0,569, n = 48), nine weeks (p = 0,990, n = 235) or two years (p = 0,348, n = 104) post treatment. In binary univariate logistic regression models the following odds ratios were obtained for being CCA positive when hookworm egg positive at baseline (n = 48, OR = 1,5; 95% CI[0,4–5,5] (trace = positive)/OR = 1,9; 95% CI[0,4–8,7] (trace = negative)), nine weeks(n = 235, OR = 1,1; 95% CI[0,3–4,6] (trace = positive)/OR = 0,5; 95% CI[0,06–3,8] (trace = negative)), and two years (n = 104, OR = 0,7; 95% CI[0,3–1,5] (trace = positive)/OR = 0,8; 95% CI[0,3–1,8] (trace = negative)) for the *S*. *mansoni* egg negative (6KK) subgroups.

### Short term CCA score dynamics

**Fig 3:**
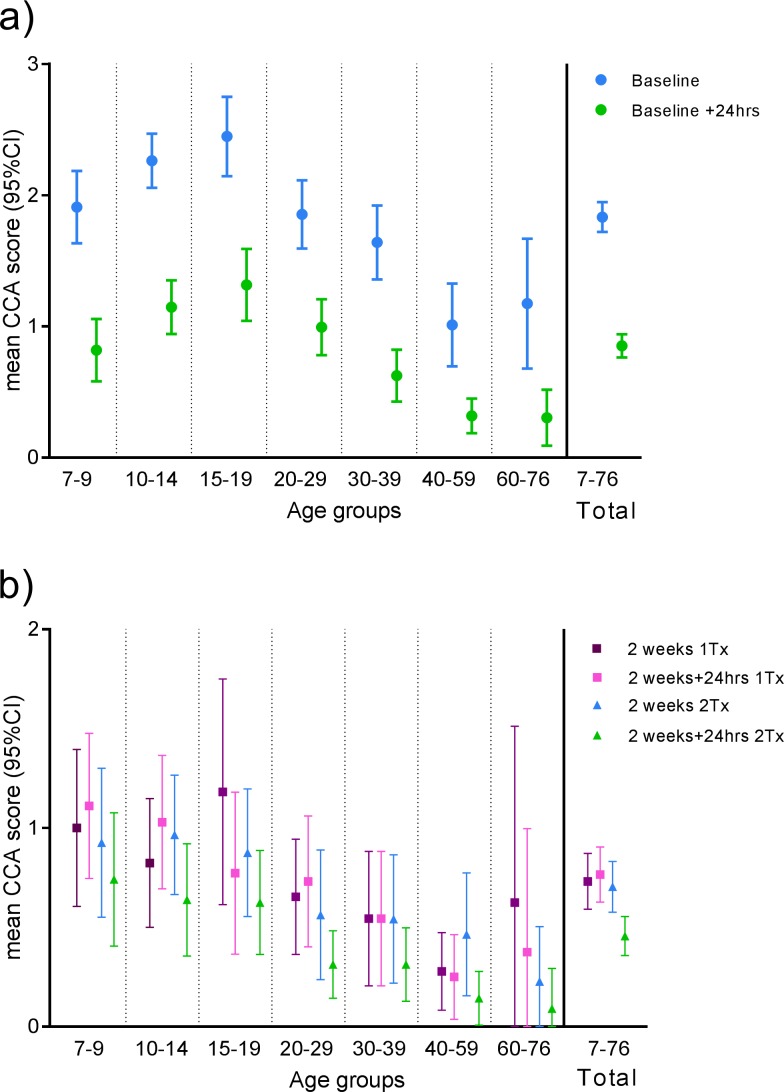
Short term CCA scores in response to treatment. The mean CCA scores with 95% confidence intervals stratified by age groupings are shown for a) baseline and baseline+24hours (n_total_ = 392) and b) two weeks and two weeks + 24hours further stratified by treatment regimen (n_1Tx_ = 147, n_2Tx_ = 157). Only individuals, who gave a urine sample at a time point (bsl and/or 2wks) and another sample at +24hrs, are included. “Trace” is included as score 0,5.

Twenty four hours after baseline 74,9% of CCA positives (n = 339) have a decline of ≥1 score unit after treatment (Table 2, Table C in S1 Table), whereas only 3,5% have an increase of ≥1 score unit. 12,4% had unchanged positive CCA scores. With respect to trace scores 5,3% and 3,2% showed a decline or increase of ≥1 score unit, respectively, whereas only 0,6% remained unchanged as trace positives. Fifteen individuals had trace positive score at baseline whereof 80% (12) were negative 24 hours later, two remained as trace positive and only one observation showed an increase in score to “1” (Table C in S1 Table). The mean CCA scores at baseline and 24 hours after treatment were significantly different (p<0.001). Mean CCA score [±95%CI] stratified by age groups for baseline and plus 24 hours is shown in Fig 3A.

**Table 2:** Change in CCA score units in response to treatment 24 hours after baseline and two weeks.

Change in CCA score	Bsl -> bsl + 24hrs	2 wks-> 2wks + 24hrs	
	n	1 Tx, n	2 Tx, n
≥ 1 ↓	254	19	34
≥ 1 ↑	12	27	7
0,5 ↓	18	13	21
0,5 ↑	11	6	9
1/2/3 → 1/2/3	42	35	20
0,5 → 0,5	2	1	8
0 → 0	53	46	58
Total	392	147	157

Horizontal arrow indicates no change in score, upwards arrows indicate a rise in score and downwards arrows a decrease in score. Only individuals, who gave a urine sample at a time point (bsl and/or 2wks) and another sample at +24hrs, are included. Non-summarised score changes can be seen in Table C in S1 Table.

The extend of daily fluctuations in measured CCA levels can be observed at two weeks where 18,8% (n = 101) of CCA positives only treated at baseline (1 Tx) had a decline in CCA score of ≥1 at plus 24 hours and 26,7% had an increase of ≥1 (Table 2, Table C in S1 Table); 35,6% had an unchanged positive score in this group. Comparably, 34,3% had a decline and only 7% had an increase in CCA score of ≥1 at 24 hours post treatment in the arm being treated at two weeks (2 Tx). In the two treatments arm 28,3% had an unchanged positive score. However, the arm only having received treatment at baseline (n = 147) show no significant difference in means at two weeks and two weeks plus 24 hours (p = 0,568). In contrast, the arm receiving a second treatment at two weeks (n = 157) has a significantly lower mean CCA score 24 hours post-treatment (p<0,001). At the two week time point 25 individuals scored trace positive in the two treatments arm and 13 responded to treatment and scored negative 24 hours later (Table C in S1 Table). Eight remained trace positive and only four showed an increase in score. In comparison seven individuals from the one treatment arm showed trace positive scores at two weeks and a negative score at two weeks +24 hours. Only one individual remained trace positive and 5 had an increased score at two weeks + 24hours. Mean CCA score [±95%CI] stratified by age groups for two weeks and two weeks plus 24 hours is shown in Fig 3B.

CCA scores at the baseline +24 hours and two week time points both reflect measurements of antigen in urine after a single treatment at baseline only. Table 3 shows the number of individuals (n) with a given score at baseline+24hrs after treatment and the corresponding score at two weeks post-treatment. Forty-three percent (n = 359) of individuals had an unchanged score from baseline +24hours to the two week time point. When considering change in score at sample level, only 36 discrepancies are observed (Table 3). Out of these score differences 19 are positive at both time points with a trend of lower score at two weeks, leaving only 17 (5%) observations positive at baseline +24hrs after treatment, which are negative at two weeks, giving an overestimation of prevalence compared to two weeks post-treatment (60% vs 55%, trace considered positive).

**Table 3:** Comparison of CCA scores at +24hrs after baseline treatment vs. two weeks.

CCA score		2 wks (n)					Total (n)
		0	0.5	1	2	3	
Bsl+24hrs 1Tx n = 359	0	102	13	25	5	0	145
	0,5	13 + **2**	5	14	2	0	36
	1	25	14 + **3**	24	19	3	88
	2	5 + **13**	2 + **6**	19 + **7**	17	4 + **2**	75
	3	**2**	0	3 + **1**	4	5	15
	Total	162	43	93	47	14	359

Light grey fill indicates an unchanged score from baseline (bsl) +24hours to two weeks (wks). **Bold** shown as “+ **n**” indicates the number for discrepant score values from baseline +24hours to two weeks on an overall sample level; for example, there are 18 observations of a score going from 2 to 0 but only 5 going from 0 to 2, meaning there is a discrepancy of + 13 in this score change category.

## Discussion

### Short timeframe CCA measures in response to treatment

In order to design the best possible interventions aiming at breaking *S*. *mansoni* transmission, availability of accurate diagnostic tools detecting live worms and thus active infection is crucial. Such tools can improve the understanding of the actual efficacy of treatment and proportion of re-infection both of which are currently based on faecal egg counts (KK). When evaluation of treatment efficacy is based on classical faecal egg counts alone, a portrayal of poor treatment efficacy may be the result as eggs produced by newly matured worms and eggs lodged in tissue and being released over a period of time, despite clearance of worms, will present as treatment failure. For antigen-based diagnostic tools, it is necessary to know whether the detected antigen is removed from circulation in response to treatment, as it is otherwise impossible to interpret whether this tool can be used to evaluate re-infection levels and treatment efficacy. To our knowledge this is the first report describing a consistently detectable decline of CCA after only 24 hours in response to praziquantel treatment in a community based study using the urine strip test. The decreased level of CCA in response to this first round of mass drug administration in this community could be observed both after baseline treatment and again at two weeks in the two treatments arm, albeit to a lesser extent quite possibly due to the already lowered worm burden. This supports the hypothesis that positive CCA measures 24 hours after treatment reflect the presence of CCA derived from immature worms, which are not susceptible to praziquantel [8], as well as from adult worms surviving PZQ treatment. A proportion of these immature worms will then have matured by the two week time point and treatment will take effect at the second administration. Murine experimental work support this view as studies have shown that CCA is detectable in liver tissue as early as two weeks post infection depending on infection dose [16, 32]. Our own observations (personal communication A. Kildemoes) show detectable CCA in mouse urine based on the POC-CCA test from 2,5 weeks post-infection depending on infection intensity. These observations point towards CCA being excreted as soon as the immature worms start feeding in the bloodstream. Our observations of consistently declining CCA short term levels in response to praziquantel treatment in the vast majority of this community sample indicate that CCA measures can bear more weight in terms of interpreting treatment efficacy, potential resistance and re-infection patterns. Repeated CCA measures in individuals could potentially be used to assess how susceptible adult worms are to praziquantel and provide information on potential up-concentration of less susceptible worm populations. The consistent decline in CCA measures was observed despite the fact that differences in clearance rate in response to treatment are to be expected due to varying initial infection intensity, daily CCA level fluctuations and host metabolism. For individual diagnosis this tool should ideally be used in combination with other diagnostic measures such as egg counts or schistosomal DNA in faecal matter in order to gain a clearer picture of the true burden of infection and related disease manifestations in the host.

### Interpretation of trace positive results; sensitivity and specificity

Both the lateral flow test used here and the POC-CCA test have showed good specificity and sensitivity for *S*. *mansoni* in large studies including sub-Saharan multi-country settings [33, 34], however the interpretation of trace measures need elucidation. More stable performance by CCA than KK on this study’s data is shown by estimated sensitivities and specificities for both diagnostic methods fitted in a latent markov model with no gold standard assumed and published in Koukounari *et al*. (2013) [21]. Traditionally calculated comparisons of CCA to KK performance can be found in Table A in S1 Supporting information. Lamberton *et al*. (2014) argues that trace measures should be considered positive based on a study carried out in children from an endemic area in Uganda comparing a 6KK as standard with a single urine sample tested with POC-CCA [35]. The data presented here support this view as we observe a response to treatment in the majority of trace positives within 24hrs at both treatment time points (Table C in S1 Table).

The strip test used for this study has a comparable sensitivity to the POC-CCA, hence the current results point towards trace measures in POC-CCA to be considered as positives in populations comparable to the sample studied here. This is particularly relevant in high prevalence areas in a mass drug administration context as the additional drug cost and minimal praziquantel adverse effects can be perceived as acceptable even if a small proportion of trace positives are indeed *S*. *mansoni* negative. However, more knowledge on sensitivity and specificity in terms of potential false positives due to cross-reactivity mediated by other helminth infections is needed, when operating in low-endemic settings and/or when elimination is targeted and a test-then-treat approach is implemented. When testing cross-reactivity on sera and urine from people infected with parasites it is important to take both biological compartmentalisation and geographical epidemiology into account. Worms with stages feeding within the human host would logically heighten the possibility of presence of regurgitated antigens. Some of these could potentially share epitopes with CCA. Furthermore, killing of tissue dwelling or migratory stages could release antigens to the same host tissue compartments as schistosomes. This could be the case with migratory or tissue dwelling stages of parasites such as filarial nematodes, *Fasciola* spp. or in cysticercosis. The monoclonal IgG antibody, which recognise CCA from adult and immature actively feeding schistosomes and not egg-derived CCA [29, 36, 37], used in this study and in the POC-CCA, has been tested for cross-reactivity on sera from people infected with a range of parasites [15, 38]. However, the sample sizes for each parasite is very small and further studies are needed to identify to which extent false-positives occur. Here we showed that the often co-endemic hookworm infection is not likely to be a confounder for the assay as there is no association observed between hookworm and CCA positivity (irrespective of traces being interpreted as positive or negative). Recently, caution in terms of interpreting test read-outs of the POC-CCA when used in pregnant women has been raised [39]. Pregnant women may present with a changed Lewis-X moiety level in urine and this could possibly increase the background. However, while more research is needed in order to elucidate potential cross reactions, current data support the application of the available CCA tests into the existing schistosomiasis epidemiology and diagnostics toolbox [40, 41]. Application of the tests should be combined with guidelines for use and interpretation of test results, which are suited for mass drug administration based control programmes and individual diagnosis in test-then treat approaches, respectively. Both the current study based on urine strip CCA test in a community sample and POC-CCA studies in children even in a low endemic sub-Saharan settings have presented less variability and better or comparable sensitivity of CCA than a single KK alone [21, 42–44], despite the fact that daily CCA fluctuations exist [42].Furthermore, application in combination with other tools in a migrant and traveller context with histories of exposure to fresh water in *S*. *mansoni* endemic regions is also relevant as it opens up for earlier detection of infection and better clinical management [45]. Potential discrepancies in interpretation of colour reaction development is particularly relevant in the context of trace/no trace scoring in terms of inter-reader variability [26].

### Expanded applicability of CCA measures

For monitoring treatment efficacy purposes, it is relevant to compare the prevalence outcomes obtained at baseline+24hrs and the two weeks after treatment time points. An overall prevalence deemed similar at these two time points equals a logistical advantage for screening strategies. 43% of individuals, who gave urine samples at both time points (n = 359), had an unchanged score (Table 3). Only 10% of observations differ at overall sample level at the two time points in terms of intensity of infection. The majority of these discrepant scores are positive at both time points, which doesn’t affect prevalence estimations at population level. The remaining observations with a positive score at baseline+24 hours and score 0 at the two week time point constitute only 5% resulting in a marginal overestimation of prevalence. The finding that a prevalence estimate made 24hrs after baseline treatment based on a single urine strip CCA test presented only a small over-estimation of prevalence compared to the two week time-point as result of a single treatment, provide prospects of using this tool as a cost-effective prevalence screening method [46]. A team could gain the same information on a single overnight field site visit compared to deploying a team a second time. There might also be positive compliance outcomes of such a strategy as people are already on site combined with the more socially acceptable and ease of delivery urine sample compared with faecal sampling. Furthermore, this would provide basis for administration of a second dose of praziquantel to individuals with residual high scores to maximize the efficacy of treatment either immediately and/or administered two to three weeks later by a community worker in order to allow immature worms to mature and become drug susceptible.

### Concluding remarks

A consistently measurable decline in CCA levels is seen in urine already 24 hours after both a single and a second praziquantel treatment in a community sample. The decline in CCA in response to treatment occurs in the majority of individuals even for trace measurements, which supports that trace should be considered positive in populations and transmission zones comparable to this study. These observations inform use of and provide weight to further interpretation of CCA based diagnostics and support extended applicability of the CCA based tools for *S*. *mansoni* in control programmes and on individual diagnostics level.

## Supporting information

S1 Checklist (DOCX)

S1 Table (DOCX)

S1 Supporting information (DOCX)

S1 Dataset (XLSX)
