# Supplementary material for: Rapid clearance of Schistosoma mansoni circulating cathodic antigen after treatment shown by urine strip tests in a Ugandan fishing community – Relevance for monitoring treatment efficacy and re-infection
Source: PLoS Negl Trop Dis. 2017 Nov 13;11(11):e0006054. doi: 10.1371/journal.pntd.0006054 (PMC5703575; doi:10.1371/journal.pntd.0006054)
Supplement: S1 Table — (DOCX) [file pntd.0006054.s002.docx]

| *S. mansoni* |  | **Baseline** |  | **9 weeks **** | | **2 years** | |  |
| --- | --- | --- | --- | --- | --- | --- | --- | --- |
| KK detection | 1 tx | 2 tx | total | 1 tx | 2 tx | 1 tx | 2 tx | |
| Prevalence (n) | 89% (239) | 88% (206) | 89% (445) | 48% (228) | 31% (200) | 67% (186) | 68% (160) | |
| GM EPG 6KKs (n) | 264 (213) | 241 (182) | 253 (395) | 20 (109) | 10 (61) | 40 (124) | 43 (108) | |
| Median (EPG) | 250 (239) | 244 (206) | 250 (445) | 0 (228) | 0 (200) | 10 (186) | 10 (160) | |
| Range (EPG) | 3 - 6527 | 3 - 5907 | 3 – 6527 | 3 - 1827 | 3 - 213 | 3 – 3330 | 3 – 4333 | |
| GM EPG KK1.1 (n) | 341 (180) | 329 (162) | 335 (342) | 78 (45) | 40 (17) | 91 (86) | 104 (78) | |
| Median (EPG) | 180 (235) | 180 (206) | 180 (441) | 0 (224) | 0 (196) | 0 (185) | 0 (160) | |
| Range (EPG) | 20 - 9340 | 20 - 7940 | 20 – 9340 | 20 - 2380 | 20 - 200 | 20 - 3560 | 20 – 2740 | |
| GM EPG KK1.2 (n) | 336 (179) | 309 (161) | 323 (340) | 55 (61) | 35 (28) | 100 (76) | 117 (73) | |
| Median (EPG) | 190 (236) | 190 (206) | 190 (442) | 0 (224) | 0 (195) | 0 (185) | 0 (160) | |
| Range (EPG) | 20 - 9820 | 20 - 8680 | 20 - 9820 | 20 - 2660 | 20 - 180 | 20 - 3100 | 20 – 2860 | |
| GM EPG KK2.1 (n) | 322 (184) | 339 (151) | 330 (335) | 55 (52) | 36 (24) | 86 (78) | 135 (65) | |
| Median (EPG) | 200 (233) | 180 (202) | 200 (435) | 0 (219) | 0 (191) | 0 (182) | 0 (151) | |
| Range (EPG) | 20 - 6360 | 20 - 7000 | 20 - 7000 | 20 - 1420 | 20 - 300 | 20 - 2360 | 20 – 5060 | |
| GM EPG KK2.2 (n) | 323 (180) | 345 (149) | 333 (329) | 64 (51) | 35 (22) | 84 (75) | 125 (69) | |
| Median (EPG) | 210 (234) | 200 (202) | 200 (436) | 0 (219) | 0 (192) | 0 (182) | 0 (151) | |
| Range (EPG) | 20 - 6740 | 20 - 7600 | 20 - 7600 | 20 - 1280 | 20 - 260 | 20 - 1860 | 20 – 5400 | |
| GM EPG KK3.1 (n) | 363 (175) | 290 (154) | 327 (329) | 54 (55) | 47 (24) | 108 (74) | 96 (60) | |
| Median (EPG) | 170 (228) | 180 (198) | 180 (426) | 0 (212) | 0 (189) | 0 (175) | 0 (142) | |
| Range (EPG) | 20 - 7440 | 20 - 4500 | 20 - 7440 | 20 - 3080 | 20 - 340 | 20 - 2220 | 20 – 4940 | |
| GM EPG KK3.2 (n) | 360 (178) | 289 (156) | 325 (334) | 55 (54) | 39 (24) | 108 (71) | 111 (57) | |
| Median (EPG) | 180 (228) | 180 (198) | 180 (426) | 0 (212) | 0 (188) | 0 (175) | 0 (142) | |
| Range (EPG) | 20 - 7020 | 20 - 4420 | 20 - 7020 | 20 - 2720 | 20 - 280 | 20 - 1940 | 20 – 5000 | |

**Supplementary tables:**

**Table A:** **Eggs per gram (EPG) per Kato-Katz (KK) slide stratified by treatment regimen and sample time.**
Prevalence in Musoli village is shown as observed by 6KK slides and for each first – sixth slide. Sample size is shown in brackets. Range and geometric mean (GM) EPG is showed for positive samples only. At 9 weeks significantly fewer *S. mansoni* eggs in stool are found when having received two treatments compared to one both at individual slide level (p≤0.002) and the total sample (p<0.001). At baseline (p=0.553) and at 2 years (p=0.716) no significant differences between treatment regimens are observed.

**Table B: CCA scores at baseline, nine weeks at two years related to mean EPG measures in intensity categories**

|  |  | *S. mansoni* egg intensity categories | | | |
| --- | --- | --- | --- | --- | --- |
|  | CCA score | Negative (n) | Low (n) | Moderate (n) | High (n) |
| Baseline | 0 | 35 | 33 | 8 | 3 |
|  | 0.5 | 4 | 12 | 1 | 1 |
| n = 443 | 1 | 7 | 28 | 25 | 5 |
|  | 2 | 3 | 17 | 50 | 45 |
|  | 3 | 0 | 13 | 27 | 126 |
| 9 weeks | 0 | 151 | 34 | 0 | 2 |
|  | 0.5 | 30 | 13 | 1 | 0 |
| n = 395 | 1 | 44 | 49 | 4 | 0 |
|  | 2 | 11 | 39 | 5 | 1 |
|  | 3 | 0 | 7 | 2 | 2 |
| 2 years | 0 | 52 | 28 | 0 | 0 |
|  | 0.5 | 11 | 8 | 0 | 0 |
| n = 320 | 1 | 36 | 61 | *7* | 1 |
|  | 2 | 3 | 39 | 16 | *5* |
|  | 3 | 2 | 15 | 21 | 15 |

The number of individuals (n) from this community sample with given CCA scores at baseline, nine weeks at two years related to mean KK (6 slides) EPG measures in intensity categories (low = 1-99; moderate = 100-399; high = ≥400). Data not stratified by treatment arm. Using egg intensity categories on ordinal scale 0, 1, 2, 3, nonparametric correlations are significant; bsl (p<0.001, n=443, *ρ*=0.703), nine weeks (p<0.001, n=395, *ρ*=0.493), two years (p<0.001, n=320, *ρ*=0.639).

**Table C: Short term CCA score changes at individual level in response to treatment at baseline and 2 weeks stratified by treatment regimen.**

|  |  | + 24 hours (n) | | | | | Total (n) |
| --- | --- | --- | --- | --- | --- | --- | --- |
|  | CCA score | 0 | 0.5 | 1 | 2 | 3 |  |
| Baseline | 0 | *53* | 10 | 7 | 0 | 0 | 70 |
| 1Tx | 0.5 | 12 | *2* | 1 | 0 | 0 | 15 |
| n = 392 | 1 | **34** | 6 | *12* | 3 | 0 | 55 |
|  | 2 | **37** | **14** | **33** | *14* | 2 | 100 |
|  | 3 | **21** | **6** | **44** | **65** | *16* | 152 |
|  | Total | 157 | 38 | 97 | 82 | 18 | 392 |
| 2 weeks | 0 | *46* | 2 | 14 | 4 | 0 | 66 |
| 1Tx | 0.5 | 7 | *1* | 4 | 1 | 0 | 13 |
| n = 147 | 1 | **9** | 6 | *22* | 6 | 0 | 45 |
|  | 2 | **3** | 0 | **3** | *9* | 2 | 17 |
|  | 3 | 0 | 0 | **1** | **3** | *4* | 8 |
|  | Total | 65 | 9 | 44 | 23 | 6 | 147 |
| 2 weeks | 0 | *58* | 6 | 3 | 1 | 0 | 68 |
| 2Tx | 0.5 | 13 | *8* | 3 | 1 | 0 | 25 |
| n = 157 | 1 | **12** | 8 | *12* | 2 | 0 | 34 |
|  | 2 | **4** | **2** | **12** | *8* | 0 | 26 |
|  | 3 | 0 | **1** | **1** | **2** | 0 | 4 |
|  | Total | 87 | 25 | 31 | 14 | 0 | 157 |

The number of individuals (n) with given CCA scores 24 hours after treatment at baseline and 2 weeks (stratified by treatment regimen). No change in score is shown in *italics*, increase of ≥1 score unit is underlined (light grey) and decrease of ≥1 is shown in **bold** (dark grey).
