# Supplementary material for: Rapid clearance of Schistosoma mansoni circulating cathodic antigen after treatment shown by urine strip tests in a Ugandan fishing community – Relevance for monitoring treatment efficacy and re-infection
Source: PLoS Negl Trop Dis. 2017 Nov 13;11(11):e0006054. doi: 10.1371/journal.pntd.0006054 (PMC5703575; doi:10.1371/journal.pntd.0006054)
Supplement: S1 Supporting information — (DOCX) [file pntd.0006054.s003.docx]

**CCA urine strip performance**
The proportion of true positives (sensitivity) and true negatives (specificity) were calculated based on six KK slides as standard for the three time points, where both urine and stool samples were obtained (suppl. table 4). Sensitivity and specificity were also calculated based on an arbitrary combination standard of the available data from up to six KK slides and urine strip CCA score >0.5 (trace considered negative). For these arbitrary standard calculations a 100% specificity of both KK and the lateral urine flow CCA strip are assumed. In this context detection of eggs is the basis of all calculations; hence lower specificity does not equal worse performance for tests detecting other diagnostic parameters, here CCA, as seen when using a combined standard. The table below shows the sensitivity and specificity for the first KK slide, the urine strip CCA and a combination of the two measures. Furthermore, sensitivity and specificity are calculated for urine strip CCA trace score (0.5) considered both negative and positive. A combination of one KK and a urine strip CCA measure was consistently more sensitive than either measure used alone. The most pronounced difference in sensitivities between a single KK slide and the urine CCA strip was observed at nine weeks.

|  |  |  | **6KK** | | | **6KK+CCA>0,5** | | |
| --- | --- | --- | --- | --- | --- | --- | --- | --- |
|  | **Detection method** | **Trace** | **Bsl (n)** | **9wks (n)** | **2yrs (n)** | **Bsl (n)*** | **9wks (n)** | **2yrs (n)** |
| **Sensitivity (%)** | KK 1.1 | none | 87.5 (441) | 36.9 (420) | 71.0 (345) | 85.3 (441) | 27.9 (420) | 60.3 (345) |
|  | Urine strip CCA | neg | 85.3 (443) | 68.6 (395) | 83.3 (320) | 85.6 (443) | 76.6 (395) | 86.0 (320) |
|  | KK 1.1+Urine strip CCA |  | 93.9 (441) | 72.6 (420) | 90.5 (345) | 94.0 (441) | 79.3 (420) | 91.9 (345) |
|  | Urine strip CCA | pos | 88.9 (443) | 77.4 (395) | 87.0 (320) | 89.1 (443) | 83.2 (395) | 89.1 (320) |
|  | KK 1.1+Urine strip CCA |  | 95.4 (441) | 79.1 (420) | 91.3 (345) | 95.5 (441) | 84.2 (420) | 92.6 (345) |
| **Specificity (%)** | KK 1.1 | none | n/a | n/a | n/a | n/a | n/a | n/a |
|  | Urine strip CCA | neg | 61.2 (443) | 76.7 (395) | 60.6 (320) | n/a | n/a | n/a |
|  | KK 1.1+Urine strip CCA |  | 80.0 (441) | 78.6 (420) | 64.0 (345) | n/a | n/a | n/a |
|  | Urine strip CCA | pos | 71.4 (443) | 64.0 (395) | 50.0 (320) | 89.7 (443) | 83.4 (395) | 84.9 (320) |
|  | KK 1.1+Urine strip CCA |  | 72.0 (441) | 67.0 (420) | 54.4 (345) | 90.0 (441) | 85.4 (420) | 82.5 (345) |

**Table A: Sensitivity and specificity of one KK slide, urine strip CCA, and a combination of one KK slide and a urine strip CCA over time.**

*one observation of CCA = 0.5 which scored 1 at bsl+24hrs was included as CCA positive. Not applicable (n/a) is indicated, where the detected measure’s specificity inherently is 100% because it is an intricate part of the standard. KK1.1 refers to the first slide of the first KK taken.

Binary univariate logistic regression show the following odds ratios for being *S. mansoni* egg positive (6KK) when CCA positive at baseline (n=443, p<0.001): OR=19.9; 95% CI[9.9-39.8](trace = positive) and OR=22.6; 95% CI[10.7-47.8] (trace = negative), at nine weeks (n=395, p<0.001): OR=6.1; 95% CI[3.8-9.6] (trace = positive) and OR=7.1; 95% CI[4.6-11.3] (trace = negative), and at two years (n=320, p<0.001): OR=6.7; 95% CI[3.9-11.7](trace = positive) and OR=7.7; 95% CI[4.5-13.1] (trace = negative). Binary multivariate logistic regression models controlled for hookworm, gender and adult (>15) /child status (≤15) give odds ratios at baseline (n=434, p<0.001): OR=14.5; 95% CI[6.8-30.9] (trace = positive) and OR=16.8; 95% CI[7.4-38.3] (trace = negative), at nine weeks (n=394, p<0.001): OR=5.9; 95% CI[3.6-9.5] (trace = positive) and OR=6.7; 95% CI[4.1-10.7] (trace = negative) and at two years (n=320, p<0.001): OR=4.3; 95% CI[2.4-7.7] (trace = positive) and OR=4.9; 95% CI[2.8-8.7] (trace = negative).
